# Quality of Life Philosophy II: What is a Human Being?

**DOI:** 10.1100/tsw.2003.110

**Published:** 2003-12-01

**Authors:** Søren Ventegodt, Niels Jørgen Andersen, Maximilian Kromann, Joav Merrick

**Affiliations:** The quality of Life Research Center, Copenhagen, Denmark

**Keywords:** quality of life, QOL, philosophy, human development, holistic medicine, public health, Denmark

## Abstract

The human being is a complex matter and many believe that just trying to understand life and what it means to be human is a futile undertaking. We believe that we have to try to understand life and get a grip on the many faces of life, because it can be of great value to us to learn to recognize the fundamental principles of how life is lived to the fullest. Learning to recognize the good and evil forces of life helps us to make use of the good ones.To be human is to balance between hundreds of extremes. Sometimes we have to avoid these extremes, but at other times it seems we should pursue them, to better understand life. With our roots in medicine, we believe in the importance of love for better health. The secret of the heart is when reason and feelings meet and we become whole. Where reason is balanced perfectly by feelings and where mind and body come together in perfect unity, a whole new quality emerges, a quality that is neither feeling nor reason, but something deeper and more complete.In this paper, we outline only enough biology to clarify what the fundamental inner conflicts are about. The insight into these conflicts gives us the key to a great deal of the problems of life. To imagine pleasures greater than sensual pleasures seems impossible to most people. What could such a joy possibly be? But somewhere deep in life exists the finest sweetness, the greatest quality in life, the pure joy of being alive that emerges when we are fully present and life is in balance. This deep joy of life is what we call experiencing the meaning of life.

## INTRODUCTION

The human being is a complex matter. Many people believe that just trying to understand life and what it means to be human is a futile undertaking. There are so many things in life, so many words, so many aspects to comprehend. But of course we have to try to understand life and get a grip on the many faces of life. It can be of great value to us to learn to recognize the fundamental principles of how life is lived to the fullest. Learning to recognize the good and evil forces of life helps us to make use of the good ones.

To be human is to balance between hundreds of extremes. Sometimes we have to avoid these extremes, but at other times it seems we should pursue them, to better understand life. Whatever we do on the surface of life, the crucial point of living is to be in balance at a fundamental level, that is, always live by our inner purpose.

In another paper in this series we will explore the question of what the living state is, in an attempt to understand the connection between physical illness and a poor quality of life, but in this paper, we outline only enough biology to clarify what the fundamental inner conflicts are about. The insight into these conflicts gives us the key to a great deal of the problems of life.

## A YOUNG WOMAN IS OFF TO A DANCE

We are all familiar with situations where we want to do something that we know to be foolish. A battle is fought inside of us. Sometimes reason wins, sometimes that other part of us does: intuition, feelings, sexual passion, or simply the temptation to eat just one more cookie. Every time we make a decision that is not right for us, we create a chaos in our lives for which we will eventually be held accountable. If we are too emotional and let ourselves be swept away, there will often be a price to pay later: We wasted our time and energy, we were used, we did not get our hearts into it. For example, an unchecked sexual desire can lead to pregnancy or venereal disease or erratic eating habits can lead to overweight and dental problems.

If a young woman goes to a dance and feels the urge to be with a man, she can choose to do so. Later she will have to deal with all the problems this might lead to: a bad relationship with him later, fighting with her parents, maybe an unwanted pregnancy, or a venereal disease. Or she can be a nice girl, who does not fool around. In that case she will stay home and may start feeling sorry for herself that she never has any fun, like all the not-so-nice girls do.

Striking the right balance between emotion and reason is obviously crucial. If a woman becomes too easygoing and loose, it could very well mean the end of her good life. Socially and existentially, it could ruin her life. Particularly if she goes to extremes and throws herself into the arms of a succession of men, who also have many changing partners, because they too, are unable to maintain a normal relationship. Often, the price paid is emotional and sexual defeat, loneliness, and fear — and perhaps even AIDS. Many women prefer the other extreme, controlling themselves, never letting go. Perhaps she will live her whole life without ever knowing real sexual pleasure or satisfaction. In one sense this niceness is deadly, because denying our bodies and our emotions makes us cold, remote, rigid, and barren. To live only through reason is no better than living only through the feelings and sensations of the body.

The woman who knows how to balance sense and sensations, who goes to a dance to find the one she will truly love, is the woman who will know love. This is the secret of the heart: When reason and feelings meet, we become whole. Where reason is balanced perfectly by feelings and where mind and body come together in perfect unity, a whole new quality emerges, a quality that is neither feeling nor reason, but something deeper and more complete.

The reason in the brain is a local thing and the feelings in the body, such as sexual desires, are also local. But where reason and emotion come together we find the whole human being. True love. Living with your heart is living in wholeness.

If the woman becomes pregnant, she may be in doubt whether or not to have the baby. Her feelings want to have the baby, but her reason, influenced by reactions and expectations of other people, might prefer an abortion. If reason prevails over feelings she decides to have the fetus removed, but then she might spend the rest of her life missing that child, because in spite of everything, having the child would have been the right thing to do, even under circumstances less than ideal.

The only way to find the right answer is to ask your heart. To seek in the depth of your own being for the right answer[1]. Obviously, we also need to think things through, so that reason is also part of the process. Equally obviously, we must let our feelings have their say. But the final decision must not belong just to the emotions, nor just to reason. It must follow the whole, the intuitive knowledge of life, the heart.

## LOVE AND MAKING DREAMS COME TRUE

What does our little love story tell us about being human? The young woman falls passionately in love with a young man. She dreams about him and wants to spend all her waking hours with him. If he has the same dreams, then the two of them can get together and start sharing a home and things just work out. If they even find a shared set of values, then they will hopefully be happy.

Happiness derives from dreams that come true. Small, modest dreams and a pretty new dress just do not cut it. Even if, in our minds, we are completely free to choose our own values — clothes, jewelry, expensive cars — it is not until we choose to strive for what our hearts want at the deepest level that we become truly happy. Otherwise, we will be no more than content to have reached our superficial goals. For life to succeed, our head needs to recognize what the whole — the heart, or our deep self — wants. Only when we know what we truly want then we are able to realize the good life. What are those dreams about the good life, dreamt deep within our souls?

These big dreams can be about many things, but they all share a certain intense and valuable quality. It is the dream about the one and only love, the dream about the genuine, warm, and close friendship and the dream about raising a family. There is the dream of expressing life through artistic creativity. The dream about the good work, where we use ourselves to the fullest and become extremely good at what we do and really accomplish something. There is the dream of a pure, spiritual life. The dream of making a difference and making the world a better place or the dreams of experiencing the great outdoors, of being one with nature, of the great connection between man and his surroundings.

It is of little significance, which dreams we seek to realize. Since, in one sense, life is really terribly banal. It almost always comes down to the same things, the we need to feel good about ourselves, about other people, and the need to do things that we really enjoy. If we look at life with our hearts, it is all about the love of being, the love of our own existence, and everything around us.

Why is love so difficult for so many of us, the love of life and of each other? When we are young it is so easy to fall in love, but this is merely the projection of pictures of love onto another person, without any real contact or dialog with that person. But deep, persistent, and glowing love seems to be quite rare. As we see it the human being is, by nature, meant to love, so it really should be easy. In practice, however, it is difficult because we are so easily caught up in our own limited and rather unloving view of the world. When our values are not rooted in our existence, they take on a life of their own. Our existence becomes fragmented. Life gets to be about all kinds of little joys and temptations (the local pleasures), detached from the greater context that exists in our whole and is about love.

## THE DIALOG BETWEEN LIVING CELLS

Let us try and plunge into the reality of biology, into the deep recesses of existence and ask: What does it mean to be human?[2] Who am I? What am I? What does it mean to be alive?

Our lives consist of billions of small living creatures that communicate with each other and the outside world: the cells. The cells are tiny little animals that are so good at communicating that the distance between them practically vanishes. Biologically speaking, human beings are highly organized blobs of slime. This puts us on an equal footing with all other big, living creatures on Earth, the trees, the fungi, and the animals. Looking into a microscope, we will find that every little piece of us is a crawling and creeping heap of amoebas, all wandering around among each other. They all know exactly what to do, when and where, wherever they are. This is why it is possible for such a gigantic colony of cells, a human being, to exist.

When we observe one another, we prefer to imagine that we are firm and stable structures. Many people think of cells as firm little building blocks, like bricks in a wall. But this is not the case, as we realize when we look into the microscope. There is very little firmness in the colonies of cells. The whole enormous order we can observe in an organism, in some ways, is an illusion, because cells die all the time and are endlessly replaced by others. The order exists only due to continuous, dynamic processes.

The only permanent thing in our biology is the recipe that specifies a human being[3]. The cells read this recipe together. They realize it in the organism, thanks to their incredible communication skills through which they all find out exactly what to do, when and where. This fluidity makes life very sensitive to imbalance and disturbance. As we shall see in the next papers this gives us the key to understanding most diseases.

Unfortunately this fundamental concept of man being formed by cells communicating and sharing a “informational master-plan” of the human being is not supported by empirical evidence, neither is it in conflict with fact; the problem we face here is the most fundamental problem in the interface between philosophy and science: the problem of consciousness and perception known as the hermeneutic problem. Our philosophy of life is the framework we use to interpret reality; it is not something we can conclude from our perception of reality. Naive inductionism is a rare philosophical position these days; most philosophy of science seems to agree that our preconceived ideas — our scientific paradigm with the famous Kuhn’ian concept — are forming our perception, interpretation, and understanding of the world. So this concept of biological information embedded in this paragraph is one of many possible ways of looking at man. We believe it to be of value as it implies a perspective of human growth and development of utmost importance for our patients, as we shall see.

## A SELF IS BORN

During the embryonic stage, as we develop into humans, it all begins with the reading of the recipe. One single cell, the fertilized egg, divides itself again and again. When the dividing cells reach a certain number, they coordinate their decisions as to which cells are going to turn into what: skin, bone marrow, liver, nervous system, etc. This is done in a highly organized way that lays out the embryo in exactly three layers of cells, as found in very primitive organisms such as jellyfish. Later, a regular fish embryo emerges with gill arches, etc. As the embryo develops, we see features from toads, mammals, and, finally, humans. A billion years of evolution played through in just 3 months. More than anything else, what living cells know is how to communicate. When the cells inside of me communicate with each other, they do it so well that they practically melt into one big lump or the person that is called “me”.

Actually, there are two selves: The ego, which is part of the mind and who we think we are, and the deep self, who we are meant to be by nature, to be implemented into a human being by all the cells' continuous communication with each other[4].

The fact that the colony is not perfectly unified is a crucial point that makes room for disease and ultimately death. We can understand death as the breakdown of the colony, the loss of wholeness. Since, in reality, we are not perfectly organized, one could say that we are all somewhere between being completely broken or fragmented, that is, dead, and completely coherent and whole, that is, alive. The latter state occurs when we live completely in accordance with our deep self and human destination. The more coherent and whole we are, the better the lives of the little cells can pull in the same direction. The happier we feel, the more meaningful our lives. The clearer we can manifest our will to live, the better will be the sum of all the cells' common desire to live.

Let us return to the subject of love. When we meet another person we are, by nature, equipped with faculties for really loving this person, being close, communicating, being friends, lovers, comrades, coworkers, and more. Deep in our lives, this is what we really know how to do. Creating closeness through communication, which by the way is not a bad definition of love. When looking deeply into our biology we are, in fact, made to realize communication, closeness and love. We were born to love.

As we all know, however, loving is the hardest thing to do. A person's ability to love expresses how coherent and whole he or she is. As we mature and develop as persons, we rediscover the coherence and wholeness that makes us capable of loving. That this process also improves our health is an important point, as we shall see later. Just being able to love *oneself* is terribly difficult, but a prerequisite for being able to give love to others. Being on good terms with ourselves, maintaining a good relationship with ourselves — so to speak — is of the utmost importance if we want to create coherence and wholeness in our lives.

## THE RELATIONSHIP WITH ONESELF

The human animal is a complex creature. This is no more evident than in our ability to experience inner conflict which, apparently, animals are not bothered by. We can hate ourselves, be at war with ourselves, so that our "relationship with ourselves" is very poor. This relationship may deteriorate to the point where people become estranged from themselves and end up feeling that life is completely empty, devoid of purpose or meaning[5]. Life no longer has any value. If we do not accept or like ourselves or our own existence, chances are that we will not be of any value to ourselves, nor to the people around us. If, on the other hand, our relationship with ourselves is good, we love ourselves and are at peace with ourselves.

The problems we have with ourselves turn up when, for instance, we dart around, not knowing what we really want. When we do not know our own deep dreams and yearnings and, hence, allow ourselves to be distracted by all kinds of superficial and meaningless temptations that help us forget the emptiness and harshness of life for just a little while. And when we consider taking our own life (as approximately 1 in 20 Danes do at times, according to our survey on quality of life in Denmark), it is because we have fundamental problems with the meaning of life, with placing ourselves in life's greater context.

Our peculiar tendency to have inner conflict may be explained by our having two selves: the ego in the mind and the self in the organism, the greater whole or a superficial self and a deep self. The superficial self, the ego, derives from identifying with all the things we have learned about ourselves. Everything that parents and teachers and other authority figures have told us that we are. Since we believe what we have been told, out of deep loyalty with our parents and other significant others, we cling to our egos.

The ego is an important part of our detailed inner map of ourselves and the world. I am this and that person with such and such characteristics. The ego is placed centrally on the map and helps us to focus inwardly on our lives and dreams and outwardly on the world and its possibilities. The ego's task is to make sure that we have a good individual strategy — good "personality" — that weaves together the inner dreams with the outer possibilities. Without a strong, dynamic, and sensitive personality — strategic, patient, disciplined, etc. — we cannot expect to succeed when trying to realize our big dreams in life. Thus, the ego serves a crucial function in life and cannot be done away with. However, the ego must be cultivated so as not to get in the way of life.

However, human beings are so much more than this. Our lives and our deep selves are much more complicated and amazing. Deep in the biology of humanity lies a recipe for being human; and this recipe has tremendous potentials for love and meaningful relations. But there is even more to life: Through life's complicated communication systems we are also connected to the world around us. That is why, intuitively, we know so much — not only about ourselves but also about the world at large — even if this deep knowledge is not always easily expressed.

Apparently, the big problem in life is to get this deep knowledge to work for us and to be expressed in everyday life[6]. Our ego gets in the way. Everything we have learned about life, the world, and ourselves is preventing any immediate recognition and realization of our deep self. Deep within, everyone holds truths about human nature that are unfathomable and abstract and yet, at the same time, so simple and self-evident that living by them should be the easiest thing in the world.

However, if we stick to reason and make a little sensible system out of everything we learned about the world from our parents and teachers, life may just elude us. It may seem easy to go by reason, but the result is often that we go through life like zombies. The unreflective reality of reason, in which we still believe, holds all the rules and “facts” we were taught when we were naive and impressionable children. In our opinion, this is what drives us away from real values and toward the shallow, inconsequential life, the life that revolves around meeting other people's expectations and the norms prescribed by society.

## DOING THE RIGHT THING

The good life requires us to learn to do the right thing, in every given situation, throughout our lives. To do the right thing as not really about acting according to societal or learned values, but much more about finding the natural and true values of your life within yourself[7]. This requires sensing, deep within our souls, what is right and what is wrong and living by it. When we live by our hearts and fullness, all the decisions we make will reflect both the inward and the outward life. This is the balanced or focused life, with the human being in equilibrium.

Such a life is not necessarily unproductive or vegetating, as many people mistakenly believe. When we find the balance and the deep meaning of life, the level of energy rises dramatically. The sense of joy and commitment, deriving from feeling fully and wholly alive, is beyond words.

So, within ourselves, at the deepest level, we find the source of everything valuable. The very core of life, intuition, creative intelligence, spontaneity, and also foolishness. This is where we really discover who we are and what values we want to live by. And this is where we become really valuable to ourselves and to those around us: our spouses, friends, families, workplace, society, and the world.

To be a whole person we must know ourselves, our dreams, our nature, and live these dreams to the fullest. Express ourselves, actualize our potentials, and become complete human beings. This, of course, is the very purpose of life.

But committing ourselves to the process that should take us there, as people do when they fully realize the meaning of their life at a very personal level, is something we often shy away from at almost any cost. When it comes down to it we are lazy, slack, undisciplined, messy, and without direction. It takes a high level of self-discipline and determination to make life succeed and to take advantage of all the opportunities that life offers. Most often, we prefer to think of this discipline as unattainable, rather than fighting a battle against high odds.

But there is no alternative to this battle if we want to be whole. The people who meet the challenge, who are happy because the challenge and the meaning have been found and who are doing their best under the circumstances given to them by fate, those are the people who live good lives. Those are the people who gradually solve the problems of life. Finding and recognizing meaning in our lives releases a nearly inexhaustible source of energy for our use.

## WISDOM AND LIFE COMPENCY

People are inscrutable. Our lives are great mysteries. Our flesh and blood holds pieces of eternity, for as the latest generations of living organisms, we have been uninterruptedly alive for the past 3 billion years. Life has been passed on through the cells, from generation to generation, since the origin of life. Living matter carries a memory. This everlasting memory, of the beginnings of time, is a reservoir of information for us to draw on. Within us, we have all the accumulated wisdom of the world.

This wisdom, deeply rooted in our biological matter, we call our "big suitcase", as opposed to our "small suitcase", which is all the things picked up by our reason throughout our individual lives. It is this deep wisdom that makes it possible, for all of us, to live good lives. This, however, presupposes that we believe enough in ourselves to revise the view of the world we were raised to believe in, and to replace it with our own, personal knowledge of life's great truths.

Many of the world's great religions and mystical systems have this as their central message: Trust yourself! Do not live your life in blind obedience to other people's expectations (the fact that religions can lose this message when faith is institutionalized is a different matter). Believing in oneself means expanding one's insight into life; a deeper insight into life leads to better decisions, which in turn makes for a better life[8].

Whoever rebels against external authorities and puts him- or herself in their place wins the opportunity of becoming the real experts in their own lives and living a first-class life.

## THE FIRST, SECOND, AND THIRD CLASS OF LIFE

Taken as a whole, life can be better or worse. Let us suggest the following crude, but rather useful, division of the quality of life into three fundamental classes.

Travelling life first class makes for an excellent existence (problems are mostly perceived as challenges). On life's second class, where most of us find ourselves, every day is full of problems (problems are perceived as heavy and difficult to overcome). On life's third class, people are actually succumbing to physical or mental illness, drug abuse, or some other form of misery (problems have become insurmountable).

We may say that we are *viable*, when we function well in all areas of life and travel first class through life. Viability means we are able to solve conflicts systematically and take on problems as challenges we can learn from. When problems do not make us give up, but merely obligate us even further to do our best.

In our survey on quality of life in Denmark we asked people three questions: (1) How do they feel at work? (2) How do they feel at home? and (3) How do they feel in their free time? The results showed that some feel good at work, some feel good at home, and some feel good in their free time. Likewise, some feel bad at work, some feel bad at home, and some feel bad in their free time.

Somewhat surprisingly, statistics revealed that it is the same people (those traveling first class) who feel good in all three areas; just as it is the same people (those traveling third class) who feel bad in all areas. Those who travel second class through life do fairly well in all areas, statistically speaking. People who cannot manage their lives fear being laid off at work, feel threatened by divorce at home, and fear breaking a leg when on a skiing holiday. This leads to an interesting perspective: For instance, people who try to escape a difficult love life by embracing their careers often end up losing both.

Viability presupposes an optimal view of the world, a good map of reality that is continuously brought up to date. If we maintain the good attitude to life, with persistence, patience, and humor, we get good lives. When we are open to the irrational and intuitive sides of life and see the world as full of opportunities to be mined and engaged, we get good lives.

But many people feel quite the opposite. They see themselves as having no value, as being undesirable, unlovable, untrustworthy, and unproductive. With an attitude like that it is very difficult, if not impossible, to be viable or seeing that life is a good thing.

The inferiority complexes that most of us carry around make it very hard to be in touch with our deep selves, or to even understand and accept ourselves. Deep within us lies the divinely beautiful recipe of human nature that we try to realize. This crippled self-image does not at all match our deep selves. Therefore, the bridge we try to build between our deep selves and the outer world does not turn out very well. Building this bridge is the purpose of life.

It is easy and very tempting to fill one’s life with the quality that derives from satisfying a *small* part of our existence. We may satisfy our mouths (through the joy of food), genitals (sexual pleasures), or minds (academic discussions). It is far more difficult to obtain the kind of quality of life that comes from connecting our inner lives with the world outside[9]. Only people who maintain this balance throughout life are able to lead the good, complete lives that we all carry within as a potential.

## CONFLICT BETWEEN OUR SEXUAL, SPIRITUAL, AND MIDDLE-CLASS LIVES

In his two novels, "Steppenwolf" and "Siddharta", the German writer Herman Hesse[10,11] related the problems his protagonists go through as they try to strike a balance between life's three major, competing values systems. Each has its own blissful joy, and each tends to usurp and exclude the other two.

The erotic and sensuous dimension is the first set of values. The second set is concerned with spirituality, the joy of contemplation, insight, and enlightenment. The third lies in the life of the virtuous citizen or, in our time, middle-class values: the joy of professional and domestic accomplishment or having a career and a family.

The pleasures deriving from these domains are very different and do not manifest themselves at the same time. If you think about work while having sex, it just will not work. If you think about sex while trying to seek spiritual knowledge, forget about it. If you worry about marital problems at work, you will not be able to do your best. The art of living requires attention to all three domains, but never at the same time.

It is a paradox that to understand the essence of these values, we must surrender ourselves to them, but if we indulge in just one of them we destroy the balance in our lives. We have to go to extremes to obtain full awareness, but we have to live in balance if we want to be happy. Luckily, this paradox can be solved when we fully enter into our lives and give ourselves fully to everything we do while, at the same time, balance life by continuously doing what we know deep inside is the right thing for us.

Sexual pleasure is probably the greatest sensuous pleasure known to man. But the person who becomes obsessed with chasing sexual satisfaction is lost, because such a life will never be rich and balanced. It is easy to imagine the misery that comes from leading a life that centers solely on sexual gratification.

To imagine pleasures greater than sensual pleasures seems impossible to most people. What could such a joy possibly be? But somewhere deep in life exists the finest sweetness, the greatest quality in life, the pure joy of being alive that emerges when we are fully present and life is in balance[5,6,7,8,9,10,11,12,13,14,15]. This deep joy of life is what we call experiencing the meaning of life.

## ACKNOWLEDGMENTS

This study was supported by grants from The 1991 Pharmacy Foundation, as well as by supplementary grants from Goodwill-fonden, the JL-Foundation, E. Danielsen and Wife's Foundation, Emmerick Meyer's Trust, the Frimodt-Heineken Foundation, the Hede Nielsen Family Foundation, Petrus Andersens Fond, Wholesaler C.P. Frederiksens Study Trust, Else & Mogens Wedell-Wedellsborg's Foundation and IMK Almene Fond. We gratefully acknowledge the critical scrutiny and expert linguistic assistance of Ib Ravn, Ph.D. The research was approved by the Copenhagen Scientific Ethical Committee under number (KF)V.100.2123/91.

## BIOSKETCHES

**Søren Ventegodt, MD,** is the Director of the Quality of Life Research Center in Copenhagen, Denmark. He is also responsible for a Research Clinic for Holistic Medicine in Copenhagen and is a popular speaker throughout Scandinavia. He has published numerous scientific or popular articles and a number of books on holistic medicine, quality of life, and quality of working life. His most important scientific contributions are the comprehensive SEQOL questionnaire, the very short QoL5 questionnaire, the integrated QOL theory, the holistic process theory, the life mission theory, and the Danish Quality of Life Research Survey, 1991–94 in cooperation with the University Hospital of Copenhagen and the late pediatric professor Bengt Zachau-Christiansen. E-mail: ventegodt@livskvalitet.org. Website: www.livskvalitet.org/

**Niels Jørgen Andersen, MSc,** Professor, Department of Innovation and Economic Organization, Norwegian School of Management. This department conducts research and provides teaching in central topics related to innovation, business development, management of global companies, business history, and economic organization. Research activities within the Department are related to four core subjects within the discipline: business history, cooperative organizations, business development and entrepreneurship, and finally studies of industries with a special focus on the electricity industry. He is also the dynamic chairman of the nonprofit organization Stiftelsen Holistisk Medisin Scandinavia, which aims to support the scientific development, research, and documentation of complementary and holistic medicine in Scandinavia. E-mail: niels.j.andersen@bi.no. Website: www.bi.no/users/fgl93013/

**Maximilian Kromann**, Cand.Phil in Philosophy. His main field is existential philosophy and he has read more than 1000 specialist books regarding this subject. His most important scientific contributions are the understanding of the nature of man and the preconditions for a successful self-expression. He is also the director of the Quality of life Bookstore in Copenhagen, Denmark, where he is coaching patients and customers in selecting literature that can develop their personal philosophy of life, and he is a popular speaker on his topic.

**Joav Merrick, MD, DMSc,** is Professor of Child Health and Human Development affiliated with the Zusman Child Development Center and Division of Community Health at the Ben Gurion University, Beer-Sheva, Israel and presently the Medical Director of the Division for Mental Retardation, Ministry of Social Affairs, Jerusalem and the Director of the National Institute of Child Health and Human Development. He has numerous publications in the field of child and human development, rehabilitation, intellectual disability, disability, health, welfare, abuse, advocacy and prevention. Dr. Merrick received the Peter Sabroe Child Award for outstanding work on behalf of Danish Children in 1985 and the International LEGO-Prize ("The Children's Nobel Prize") for an extraordinary contribution towards improvement in child welfare and well being in 1987. E-mail: jmerrick@internet-zahav.net. Website: www.nichd-israel.com
